# An Adaptive Prediction Target Search Algorithm for Multi-AUVs in an Unknown 3D Environment

**DOI:** 10.3390/s18113853

**Published:** 2018-11-09

**Authors:** Juan Li, Jianxin Zhang, Gengshi Zhang, Bingjian Zhang

**Affiliations:** 1 Science and Technology on Underwater Vehicle Technology, Harbin Engineering University, Harbin 150001, China; 2 College of Automation, Harbin Engineering University, Harbin 150001, China; zjx19930919@hrbeu.edu.cn (J.Z.); zhanggengshi@hrbeu.edu.cn (G.Z.); zhangbj1202@hrbeu.edu.cn (B.Z.)

**Keywords:** multiple AUVs, cooperation, adaptive prediction, target search

## Abstract

For a target search of autonomous underwater vehicles (AUVs) in a completely unknown three-dimensional (3D) underwater environment, a multi-AUV collaborative target search algorithm based on adaptive prediction is proposed in this paper. The environmental information sensed by the forward-looking sonar is used to judge the current state of view, and the AUV system uses this environmental information to perform the target search task. If there is no target in the field of view, the AUV system will judge whether all sub-regions of the current layer have been searched or not. The next sub-region for searching is determined by the evaluation function and the task assignment strategy. If there are targets in the field of view, the evaluation function and the estimation function of the adaptive predictive optimization algorithm is used to estimate the location of the unknown target. At the same time, the algorithm also can reduce the positioning error caused by the noise of the sonar sensor. In this paper, the simulation results show that the proposed algorithm can not only deal with static targets and random dynamic interference target search tasks, but it can also perform target search tasks under some random AUV failure conditions. In this process, the underwater communication limits are also considered. Finally, simulation experiments indicate the high efficiency and great adaptability of the proposed algorithm.

## 1. Introduction

In modern marine military, underwater environment reconnaissance is achieved mainly by autonomous underwater vehicles (AUVs). Due to the complexity of the underwater environment and the randomness of the target state, AUV cannot obtain all of the target information in the mission environment in advance. Therefore, in an unknown underwater environment, AUVs must have the ability to search the target adaptively and finish decision-making for movement. In recent years, the use of multi-autonomous underwater vehicles has attracted a lot of attention in such circumstances as vast ocean conditions, deep ocean operations, underwater resource exploration, marine scientific investigations, and military enemy investigations. It needs to be noted that the optimization of the strategy for target search and tracking (monitoring) of unknown areas through rational coordination of limited resources has become a key issue.

The target search methods of most AUV systems are set up based on known environmental conditions, either globally or locally. The tracks of AUV target searches are generally pre-planned offline, and they are the outcome of optimization of tracks. References [1,2,3,4] proposed a biological heuristic neural network algorithm for an unknown environment. This algorithm was suitable for the simulation of two-dimensional environments in which the target search and collision avoidance in the unknown environment was realized by using the process of transfer and attenuation of weight between the neurons constructed in the simulated environment. However, because the sonar sensor can only detect surrounding local environments, this method can only be applied to a target search in a locally known environmental condition. The method proposed by [5] was an AUV path planning method for finding the optimal path in a known environment. The method could only find the local optimal path that satisfies lowest energy consumption principle. However, it did not solve the global optimal problem for search path and search efficiency in a known environment. In order to find the globally optimized search path and to improve the efficiency of target search, references [6,7] proposed a particle swarm optimization (PSO) algorithm based on path planning and target search; Yang et al. [8] developed an improved particle swarm optimization (PSO) method. They applied the cooperative rules of the robot system into the potential field function, which was used as adaption function for PSO. The improved PSO was used to solve the AUV team’s target search problem in a underwater environment. In order to overcome the limitations of robots in the workspace, a distributed algorithm based on particle swarm optimization (PSO) was established by references [9,10,11], which could be used to find the optimization results globally. It was found that the above method has little demand for global information, thus greatly saving data storage space. However, the capabilities of such methods are very limited because the methods were only suitable for known underwater environments by introducing a fitness function or an evaluation index. Therefore, when the underwater environment was unknown in advance, this single evaluation index or fitness function could hardly complete the task. In another word, the target search and dynamic adaption of the AUV system could not be achieved in this manner under unknown underwater environment conditions. As matter of fact, the real operating environment for AUVs is often unknown and complex. Thus, the above-mentioned target search method cannot be directly used in a real underwater working environment. Furthermore, it is expected for AUV systems to have a higher target search efficiency and a good dynamic adaption capability when facing modern underwater environments. Therefore, references [12,13,14,15] proposed an improved strategy for target searches with combination options and learning reinforcement algorithms in an unknown environment. However, the learning reinforcement will reduce the dynamic performance of the robot during its operation, even though the algorithm is suitable for real-time tasks. In order to improve the dynamic performance, they also developed an evaluation method using PPSO (Parallel Particle Swarm Optimization) to solve the dynamic optimization problem. Nevertheless, this improved learning method greatly reduces the efficiency of the robot. In order to improve the efficiency of the target search, references [16,17,18,19] proposed an unscented Kalman filter (UKF) and particle filter methods to deal with the complexity and variability of target motion. By establishing a motion model for the target, the position estimation of the target is achieved by using relevant sonar data. References [20,21] used the template matching method to realize the comparison of information between the past and present, based on the optical mobility method, and combined sensor-related data to estimate the dynamic characteristics of the underwater target to achieve target searching and tracking. However, these methods could only be used to improve either target search efficiency or dynamic performance, and they were designed for the circumstances with known local information. As for the target search efficiency and dynamic characteristics of the AUV system in the global unknown environment, the reported investigation was not involved.

When facing the global unknown underwater environment, the coordinated target search was the key factor affecting the underwater environment operation and the dynamic characteristics of multiple AUV systems. Therefore, the task assignment of the target search for multi-AUV systems in complicated environments has become an important research direction. For example, references [22,23,24,25,26] proposed a method regarding collaborative target searches and task assignments in a complex environment for multiple AUV systems; references [27,28,29] used a new tracking metric combined with the coordinated map coverage feature in the monitoring environment to complete task assignments for unknown environmental target searches; Mottaghi et al. [30] set up the model for the probability distribution of the position of target from sampling points in particle cloud, which was used to coordinate the target tracking of multi-AUV system under a limited visibility situation. References [31,32,33] aims at multi-robots in a dynamic environment to collaboratively select multiple targets in a dynamic environment, and uses a computer simulation of dynamic environment changes to achieve multi-target selection of multi-robot motion behavior coordination. Such a method, along with multi-robot communication and multi-task coordination strategies, allowed for high-efficiency AUV work in target searching and tracking in complicated environments, whereas the method was only suitable in two-dimensional situations. Thus, the method was not capable of dealing with target searching and tracking for multi-AUV systems in a 3D unknown underwater environment.

In this paper, a target search of multi-AUV was conducted in the context of a three-dimensional underwater unknown environment. Considering the limits of underwater communication and the effects the AUV’s own motion, combined with the limits of the configured sonar sensor device in an unknown underwater environment, this paper is dedicated to proposing a sub-region collaborative search strategy and a target searching algorithm based on a perceptual adaptive dynamic prediction. The reality of the local environment is obtained by using the forward-looking sonar of the multi-AUV system. According to the proposed adaptive dynamic prediction target searching algorithm and sub-region collaborative search strategy, high-efficiency static target search and positioning, dynamic target search and tracking, and target search and tracking during the single AUV breaking down are realized in a completely unknown underwater environment. It is assumed that the area of target search meets the positioning accuracy requirements. The simulation experiments shown in Section 5 verify that with one or more of the static targets, the dynamic targets and the fault AUV are present, and the algorithm proposed in this paper and the hierarchical sub-region collaborative searching strategy successfully realizes the target searching and tracking for multiple AUVs in an unknown underwater environment. For a three-dimensional unknown underwater environment, the target search methods in above-mentioned literature are only beneficial for improving the efficiency of target searching and the optimization performance in either global known environment or locally known environment. These methods could not be directly used to solve the problem of collaborative target search for multiple AUV system specifically for three-dimensional unknown underwater environments, which is the main concern in this paper. Because both the random searching method and the lawn searching method are adaptive to the three-dimensional unknown environment constructed in this paper, the performance of both methods in terms of efficiency and task completion for multi-AUV collaborative target search and tracking are compared. Finally, the experimental data were used to verify and justify the feasibility of proposed method. The comparison between the proposed method and such methods that were reported in open literatures and referred in this paper are also be made. The performance of proposed method is proven to be superior to the others. Moreover, the experimental data fully demonstrate the adaptive characteristics of AUV when it fails in target searching and tracking.

This paper is organized as follows: Section 2 presents the problem description; Section 3 introduces the model establishment. Section 4 descripts the introduction of the adaptive prediction searching algorithm. The simulation experiments under various conditions are given in Section 5. Finally, the conclusions is drawn in Section 6.

## 2. Problem Description

In an unknown underwater environment, multiple AUVs cooperatively perform a target searching task. Compared to the terrestrial environment, the unknown underwater environment is more complex and more changeable, which results in difficulties of target searching in such conditions. Therefore, in order to effectively conduct a searching task in complex underwater condition, it is important to analyze the factors that may affect cooperative searching for multi-AUVs:

1. Communication restrictions: Since the AUV cannot receive GPS (Global Positioning System) signals when it operates in an underwater environment, the AUV can only rely on the inertial measurement unit and the Doppler log in the target search process, as well as the filters to navigate. No matter how advanced the communication equipment is, it cannot be guaranteed that the AUV can calibrate its current position and posture at all times. Furthermore, the environmental information that AUV has encountered will not be fed back to the mother ship in time. Therefore, the target search tasks can only be performed by relying on its equipment [34]. Considering such constraints as the bandwidth of underwater communication, the, distance and the range between individual AUVs in a three-dimensional underwater environment, multiple AUV systems can cause problems of data loss and information acceptance errors. Therefore, the impact of communication restrictions must be taken care of.

2. Visual noise and threshold: Through the AUV-configured forward-looking sonar, the observation data is affected by Gaussian noise during the measurement of the observation target. In addition, AUV observation of the unknown environment through the forward-looking sonar is also limited by the sensor’s detection distance [35]; that is, the sonar cannot observe and extract environmental features that are outside the view.

3. Movement limitation: The AUV’s own motion state will be affected by equipment such as thrusters and rudders. In an unknown underwater environment, the AUV will also be affected by unknown factors such as ocean currents and submarine topography [36]. Therefore, the influence of the AUV motion restriction features on the track planning during the AUV navigation process need to be considered.

4. Collaborative searching: In the process of performing search tasks for the AUV, the search efficiency of a single AUV is much lower than the multiple AUV system. In other words, multiple AUVs collaborative searching can ensure the redundancy of searching tasks, and complete the task reliably and efficiently. In order to better improve the efficiency of collaborative searching by multi-AUV, it is required that environmental data resources are shared between each AUV, and that the search tasks have to be assigned clearly for each AUV.

5. Obstacle avoidance: AUVs may operate in an unknown underwater environment, and it is inevitable for AUVs to encounter obstacles, which may threaten the AUV’s normal trajectory in the process of performing target searching tasks. Therefore, the AUV is expected to have the ability to avoid obstacles in a timely manner, to ensure that AUV can travel safely and reliably. Ultimately, the economic cost caused by equipment damage can be avoided and the searching task can be reliably completed.

6. Self-adapting to the environment: The underwater environment is intricate by nature, and its characteristics change frequently. Thus, it is required that the AUV can always sense the information of surrounding unknown environments, and make adjustment strategies when facing different environmental characteristics. Under limited resource allocation conditions, AUVs can complete the target searching tasks reasonably and efficiently, and finish target positioning.

Given the fact that there are numerous influencing factors that are related to unknown underwater environments and the target searching problem, under the premise of reliable communication between multiple AUVs, the AUV’s own motion limitation, the noise of the sonar sensor’s field of view, as well as the ability of collaborative autonomous searches for the multi-AUV system will be investigated, to evaluate their influence on the target search task in this paper.

## 3. Model Establishment

### 3.1. AUV Movement Model

In the intricate underwater environment, the key tasks of target search, tracking, and positioning are expected to be completed, in order to achieve the adaptive searching task. The AUV four-degrees-of-freedom constant-speed motion model $x_{t+1}=f(x_{t})$ was established to describe the form of AUV motion under water. In this paper, the updating of velocity and position follows the following formula:

(1) $$\left\{ \begin{array}{l} x(t+1)=x(t)+v_{x}\\ y(t+1)=y(t)+v_{y}\\ z(t+1)=z(t)+v_{z}\\ v=v_{c}\\ v_{x}=v_{c}\sin(\phi )\cos(\theta )\\ v_{y}=v_{c}\sin(\phi )\sin(\theta )\\ v_{z}=v_{c}\cos(\phi ) \end{array}\right.$$

where (x,y,z) represents the positioning information that is related to the time variable t; $\left(v_{x},v_{y},v_{z}\right)$ represents the velocity vector of the AUV in the global coordinate system; υ indicates the motion velocity of the AUV. $\upsilon_{c}$ is a normal constant and ϕ represents the angle between the AUV axis and the z axis of the global coordinate system. θ represents the angle between the AUV direction and the direction of the global coordinate system axis. AUV is affected by its own equipment, and its speed $\upsilon_{c}$ and corner ϕ,θ are limited.

### 3.2. Forward-Looking Sonar Model

**Figure 1:**
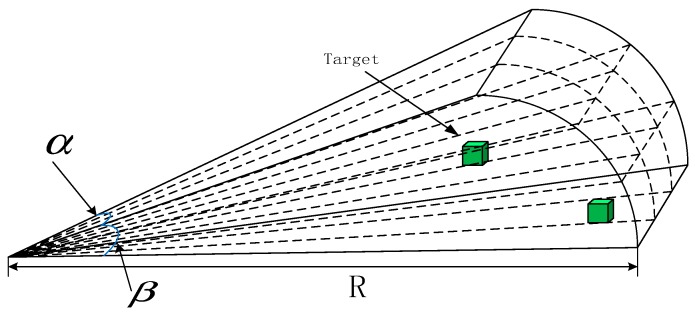
Front view sonar model.

Based on the unknown three-dimensional (3D) underwater environment, the real multi-beam active forward-looking sonar data is simulated through a mathematical model of forward-looking sonar [37] in this paper. According to the common multi-beam sonar background, the Seabeat6012 sonar is selected as the forward looking sonar of the AUV. The Seabeat6012 sonar has a visible range *R* of 150 m, a horizontal opening angle α of 120°, a vertical opening angle β of 15°, and an operating frequency of 2 kHz. The target data is obtained by using the sonar model through counting the elements of the matrix in the range of opening angle for the sonar. Moreover, whether a target is in a certain position or not in visible range is judged by the characteristics of the elements in the array of the matrix according to the model. The forward-looking sonar can be simply expressed in Figure 1.

The mathematical model to establish the relationship between the target and the forward-looking sonar limits is to determine whether the target appears within the range of the forward looking sonar, as given in Equation (2).

(2) $$\{\begin{array}{c} \frac{\left|y_{t}\right|}{\sqrt{x_{t}^{2}+y_{t}^{2}}}\leq \sin\frac{\alpha }{2}\\ \sqrt{x_{t}^{2}+y_{t}^{2}+z_{t}^{2}}\leq R\\ \frac{\left|z_{t}\right|}{\sqrt{x_{t}^{2}+y_{t}^{2}}}\leq \sin\frac{\beta }{2} \end{array}$$

where $\left(x_{t},y_{t},z_{t}\right)$ can be expressed in Equation (3):

(3) $$\{\begin{array}{c} x_{t}=x-x_{0}\\ y_{t}=y-y_{0}\\ z_{t}=z-z_{0} \end{array}$$

where (x,y,z) represents the coordinates of the target in the body coordinate system $\left(Ox_{0}y_{0}z_{0}\right)$. $\left(x_{0},y_{0},z_{0}\right)$ is the body coordinates of the forward looking sonar configured by the AUV; $\left(x_{t},y_{t},z_{t}\right)$ indicates the relative positional relationship between the target and the AUV; by determining the position relationship of $\left(x_{t},y_{t},z_{t}\right)$, it can be confirmed whether the target is in the sight of the sonar.

Because the forward-looking sonar equipped on the AUV can be easily affected by the medium of water or other external factors during the data collection process, such as data interference, the measurement of the environmental characteristics is likely to be affected. Therefore, the description of the sonar is given in Equation (4):

(4) $$y_{x-q}=\left\{ \begin{array}{ll} none & |x-q|>L\\ none & |x-q|\begin{array}{cc} in & \mathrm{Obstacles} \end{array}\\ h(x,q)+d(x,q)\zeta & |x-q|<L \end{array}\right.$$

where $y_{x-q}$ denotes the environmental feature information that is collected by the forward-looking sonar, none denotes that the feature data of the environment does not exist, *L* means the visual threshold, h denotes the sonar detection function under a no-noise condition, d denotes the relative distance between the environment feature and the sonar, and ζ is non-linear interference.

The above indicates that if the relative distance between the current visual position and the environment feature exceeds the visual range, or there is an obstacle between the sonar and the feature, the feature’s information cannot be fed back. If there is feature information in the range of sight, the noise of observation data of the feature will increase as the distance increases.

### 3.3. The Environment Model

**Figure 2:**
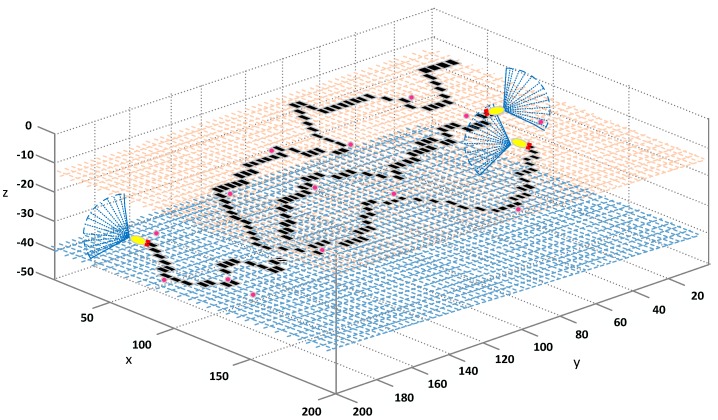
Track area coverage.

For a wide range of unknown three-dimensional sea, AUV moves from a known position, searches for targets with unknown location and quantity information, and acquires target characteristics and stores relevant data. At the same depth, a model for a large-scale underwater environment can be constructed using the grid method. The environmental matrix is established with the abscissa *x*, the ordinate *y*, and the depth coordinates. The AUV can search freely in the sea area $\left[x_{\max},y_{\max},z_{\max}\right]$. A number of n AUVs are dispatched for this task, and this task can be input at random. For any $N_{s}$ static targets and $N_{d}$ dynamic targets, the sea area is then divided into *M* × *N* grids [38] by using the grid method. The set of cells is taken as the cost matrix of the AUV track, which can be expressed as v={(i,j)|i=1,2…M;j=1,2…N}, and the length of each side of grid is set to a unit length. The pair (*i*,*j*) represents the *i*-th row and the *j*-th column in the cost matrix in a two-dimensional array. The search path cost is represented by a filling matrix value, where 1 and 0 denote the position of the sea where the AUV has and has not sailed. As shown in Figure 2, the black area represents the trajectory of the AUV sailing over a large area of the grid, and the gray and blue layers represent different layers of 3D underwater environment. Pink denotes a random target that appears in the environment. Yellow indicates the AUV, and the blue sector line is the forward-looking sonar beam.

### 3.4. Target Sports Characteristics

In the unknown complex environment, there are static targets and dynamic targets. The AUV makes different decisions by sensing different environments.

The positional information of static targets will remain unchanged at any time, so that the feature model of the static target can be described in Equation (5):

(5) $$\left[\begin{array}{c} x(k+1)\\ \begin{array}{l} y(k+1)\\ z(k+1) \end{array} \end{array}\right]=\left[\begin{array}{c} x(k)\\ \begin{array}{l} y(k)\\ z(k) \end{array} \end{array}\right]$$

If it is assumed that dynamic targets appear randomly in the environment, and they remain at a fixed water depth. That means that the AUV operates with constant angular velocity in a turning motion without considering the motion of the shaft [39]. In the rectangular coordinate system, the discrete time in the mathematical model of the moving object can be given in the form of Equations (6) and (7) respectively.

(6) $$X_{k}={\left[\begin{array}{cccc} x_{k} & {\dot{x}}_{k} & y_{k} & {\dot{y}}_{k} \end{array}\right]}^{T}$$

(7) $$X_{k+1}=\left[\begin{array}{cccc} 1 & \frac{\sin\omega T}{\omega } & 0 & -\frac{1-\cos\omega T}{\omega }\\ 0 & \cos\omega T & 0 & -\sin\omega T\\ 0 & \frac{1-\cos\omega T}{\omega } & 1 & \frac{\sin\omega T}{\omega }\\ 0 & \sin\omega T & 0 & \cos\omega T \end{array}\right]X_{k}+\left[\begin{array}{cc} T^{2}/2 & 0\\ T & 0\\ 0 & T^{2}/2\\ 0 & T \end{array}\right]\left[\begin{array}{c} w_{x,k}\\ w_{y,k} \end{array}\right]$$

where ω is the angular velocity; T is the sampling time.

In this paper, the search task is divided into the search for dynamic targets and static targets. The AUV needs to mark and store the positional information of the searched static target. For the dynamic target, it needs to predict its motion status within the sight range, and realize tracking and rounding.

### 3.5. Multiple AUV Communication Environment Analysis

When there is no communication connection among multiple AUV systems, the environmentally-oriented cognitive information is inaccurate, and the data information is incomplete. Therefore, the individual AUV will make some decisions with missing information, and the decision can be in a non-optimal state during the process of the task. As a result, AUV systems will have to spend more time and resources on performing tasks, which will seriously affect the task completion progress of the AUV system. Therefore, the impact of multi-AUV system communication environment on the efficiency of target search also needs to be considered.

AUV underwater communication relies on underwater acoustic communication. Compared with the influence of other media on the propagation speed and propagation distance of sound waves, the influence of aqueous medium on underwater communication makes signal communication between multiple AUV systems more complicated and difficult. Because the water medium has an extended degree of loss and absorption of the acoustic signal, when the communication signal between the systems is more than several tens of kilometers, the data signal bandwidth is as low as several kHz or less; if the distance between multiple AUV systems reaches several kilometers of medium distance, the data bandwidth of the communication is around the order of 10 kHz; and when communication between multiple AUV systems reaches short distances of less than a kilometer, the communication data bandwidth of approximately 100 kHz can be obtained.

In the area where multi-AUV systems collaborate to perform target searching and positioning, the cases with medium and short distances will be dealt with in this paper. Therefore, when the individual AUV shares the data information of the target with other AUVs of the multi-AUV system, the individual AUV can perform the communication within the scope of the task. Finally, the individual AUV obtains the target characteristic data reasonably and effectively from the data buffer, and all AUVs cooperate to conduct the dispatched tasks of target searching and positioning effectively.

## 4. Adaptive Prediction Search Algorithm

As pointed out, most of the current optimizations in the target searching of AUV were performed in the conditions with known or partially known environments, according to the designed global optimization routes. However, in a completely unknown environment, this kind of optimization is not flexible enough to deal with the randomness and complexity of an underwater environment. Therefore, the AUV is required to be able to make a reasonable judgment on the environment by using the external information that is sensed by the sonar in the process of target searching. Furthermore, the AUV can dynamically predict tracking information in real time and carry out adaptive target searching.

**Figure 3:**
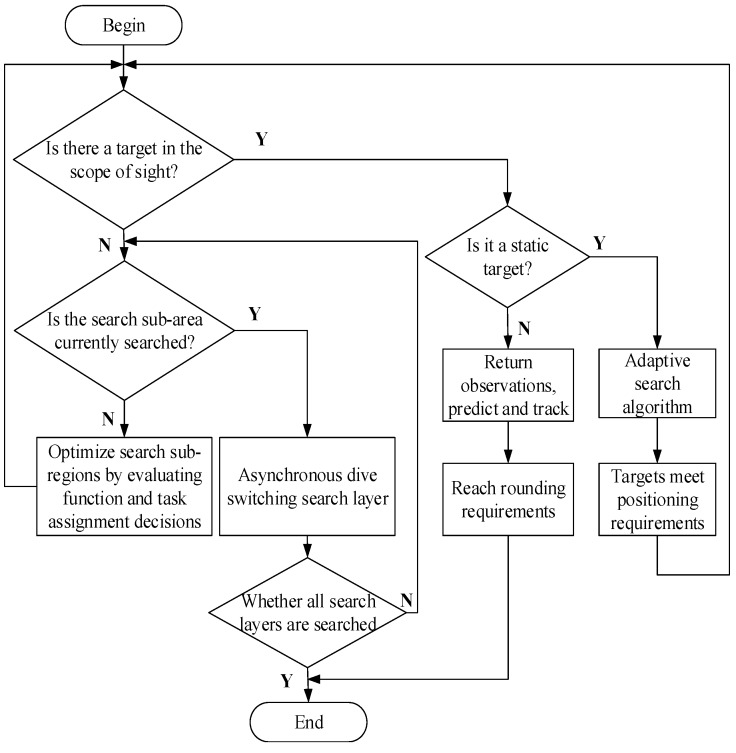
Adaptive predictive search algorithm flow chart.

The adaptive prediction in an unknown three-dimensional environment during target searching process for AUV can be roughly described in Figure 3.

### 4.1. Sub-Region Search Strategy

Due to the optimization function depending to some extent on the visual environment data in the calculation process, the multi-AUV system can only adopt the principle of searching layer-by-layer in a 3D environment. In each search layer, when there is no environmental information in the scope of an AUV, the AUV can adopt a sub-region search strategy. At the same time, the sub-areas are divided according to the characteristics of forward-looking sonar that is equipped on the AUV, and each sub-area contains an independent matrix.

First, the large-scale planar sea area is divided into several sub-search areas. The length of the rectangle side of each sub-search area is determined according to the view range of the forward-looking sonar configured by the AUV. In this paper, the side length of each sub-area is defined as being twice the length of view area. This is denoted as Equation (8):

(8) I=2∗R

where *I* denotes the side length of the rectangular sub-region, and *R* represents the field of view length of the sonar.

When the AUV performs a sub-area search strategy in the searching layer whose depth is *H*, the searching cost at each sub-area is first calculated, and it is determined whether the sub-area with the minimum cost is preset as locked, which can be denoted by Equations (9) and (10) respectively.

(9) $$\mathrm{LockArea}=\{\begin{array}{c} 0\\ 1 \end{array}\begin{array}{c} Unlocked\\ Locked \end{array}$$

(10) $$V_{i}=\min\{Vi,LockArea=0\}$$

where $V_{i}$ represents the cost for each sub-area, and the AUV prefers to consider the sub-area with the lowest search cost and unlocked as the sub-region of the searching task performed by the AUV.

After the sub-area is allocated, the AUV corresponding to the task must be numbered and recorded so that it completes the collaborative search, which can be denoted as given in Equation (11):

(11) Task(m)=i,i∈{1,2, …,n}

where *m* is the number of task area and i is the number of AUV assigned the task.

If the sub-area is locked by one AUV in a multi-AUV system, this sub-area is identified as the task sub-areas of the AUV and a target search is performed. If not, then other task sub-areas at non-locked states are re-considered. After comprehensively calculating the grid cost and the linear distance to the area, an appropriate task sub-area is selected.

After completing the search task at the layer with a depth of *h*, multiple AUVs will asynchronously switch the search layer and the switch can be expressed as follows:

(12) $$h_{x}=\left\{ \begin{array}{l} \begin{array}{ccc} 0 & & Existing\;subregions\;not\;completed \end{array}\\ \begin{array}{ccc} 1 & & All\;subregions\;completed \end{array} \end{array}\right.$$

(13) $$Z=\min(|Z_{x}-Z_{a}|)$$

where $Z_{x}$ denotes the xth search layer, and $Z_{a}$ denotes the search layer where the current AUV is located.

When AUVs perform target search in the same layer, the AUV system will record whether the current search layer has an AUV search, or it has been searched over and the AUV number of the sub-area searched is also recorded. The locked sub-areas in the same layer will have the corresponding AUV performs target search mission, and the AUV will not search for sub-areas that are not locked. Once a sub-area is locked by one AUV, then the sub-area will not be locked by others, so that collisions in the same sub-area search are avoided.

### 4.2. Adaptive Prediction Search Algorithm

Adaptive search makes AUV be able to deal with complex and unknown environments and adjust its motion. At the same time, it is required to plan the mission track the future location information online and dynamically.

Firstly the following simplified AUV motion equation is considered:

(14) $$\left\{ \begin{array}{l} X_{k+1}=f\left(X_{k},u_{k},w_{k}\right)=X_{k}+\Gamma (u_{k}+\omega_{k})\\ z_{k+1}=h(x_{k},v_{k}) \end{array}\right.$$

When the target information exists in the AUV sight, the observation equation for the target is specifically expressed in Equation (15):

(15) $$z(k)=\left[\begin{array}{l} \chi \\ \alpha \\ \beta \end{array}\right]=\left[\begin{array}{l} \sqrt{\left(x_{i}-x{\left(k\right)}^{2})+(y_{i}-y{\left(k\right)}^{2}+(z_{i}-z{\left(k\right)}^{2}\right)}\\ \arctan\frac{y_{i}-y(k)}{x_{i}-x(k)}-\alpha_{0}\\ \arctan\frac{z_{i}-z(k)}{\sqrt{(x_{i}-x{\left(k\right)}^{2})+(y_{i}-y{\left(k\right)}^{2}}}-\beta_{0} \end{array}\right]$$

where χ denotes the relative distance between AUV and the target; $\alpha_{0}$, $\beta_{0}$ denotes the directional angle of the AUV in the horizontal and vertical directions respectively α, β denote the angle between the horizontal and vertical directions of the target in the view area with respect to the AUV.

An optimization evaluation standard function [40] can be defined as given in Equation (16):

(16) $$J(x,q)=\int_{q\in v,q\notin D}||x^{\left(i\right)}-q||dq+K\int_{q\in v\cup D}dq+\zeta$$

In the above equation, *x* and *q* indicate the positional information of the AUV and the target, *v* indicates the view range of the AUV, *D* indicates the task area, *K* is an attenuation factor, and ζ is interference noise related to the relative distance. In this paper *K* = 0.3 is set.

At each time step, the noise measurement estimates of these functions are sensed and calculated by using the above-mentioned optimization function. A function approximation method can be used to estimate the unknown target function *J* at each time *k* according to Equation (17):

(17) $${\hat{J}}_{k}{}^{n}(x_{k}^{\left(1\right)},\ldots ,x_{k}^{\left(N\right)})=\vartheta_{k}^{}\phi (x_{k}^{\left(1\right)},\ldots ,x_{k}^{\left(N\right)})$$

where ${\hat{J}}_{k}$ is the estimated value of the optimization evaluation function; $\vartheta_{k}^{}$ represents the parameter estimation vector to be calculated at time k; ϕ represents the regression vector with mean value of 0.

The vector $\vartheta_{k}^{}$ can be calculated along with the least square method according to Equation (18):

(18) $$\vartheta_{k}=\arg\min\frac{1}{2}{\sum_{\ell =\ell_{k}}^{k-1}\left(J_{\ell }^{n}-\vartheta \phi (x_{\ell }^{\left(1\right)},\ldots ,x_{\ell }^{\left(N\right)})\right)}^{2}$$

The AUV predicts R candidate positions at time k+1 according to the current position at time *k*:

(19) $$x_{k+1}^{i,j}=x_{k}^{\left(i\right)}+\alpha_{k}\varsigma_{k}^{i.j},i\in \{1,\ldots ,N\},j\in \{1,\ldots ,R\}$$

where $\varsigma_{k}^{i.j}$ is a random noise with a mean value of 0 and unit variance; $\alpha_{k}$ is a positive sequence that is not greater than the current maximum speed that satisfies the conditions:

(20) $$\lim_{k\to \infty }\alpha_{k}=0\sum_{k=1}^{\infty }\alpha_{k}=\infty \sum_{k=1}^{\infty }\alpha_{k}^{2}<\infty$$

In order to ensure the convergence of the algorithm, another important choice is the expression of the sequence $\alpha_{k}$, defined in Equation (21). The typical choice of this sequence is given by:

(21) $$\alpha_{k}=\frac{c}{{\left(k+1\right)}^{\eta }}$$

where *c* is a positive user-defined constant and η∈(1,1/2). In this paper, *c* = 0.2 and η = 0.15 are set.

After calculating all feasible candidate positions, the optimal standard value is selected as the new position of the next moment of AUV:

(22) $$[x_{k}^{\left(1\right)},\ldots ,x_{k}^{\left(N\right)}]=\underset{j\in \{1,\ldots ,R\}}{\arg\min}\hat{J_{k}(x_{k}^{1,j},\ldots ,x_{k}^{Nj})}$$

Because the AUV’s own position information is accurately calculated in real time in advance, the probability error of the target position information can be reduced. Therefore, the unscented Kalman filter (UKF) [41] is used to reduce the influence of interference on the data and to prevent the accumulation of errors from failure of the algorithm. In combination with the dynamic prediction, the interference on the instant tracking data is reduced.

The above logic can be simplified as follows: when a target appears within the scope of view, a set of positions of many candidate AUVs can be generated at each time point. The ${\hat{J}}_{k}$, which provides the best estimate of the objective function, is selected, and the predicted data is taken as the new location of AUV at the next time. The prediction and selection of candidate locations is the key to the algorithm, which will influence the effective judgment of ${\hat{J}}_{k}$ and the accurate estimation of the unknown function *J*.

### 4.3. Target Location Estimation

The AUV uses the forward-looking sonar sensor to sense the external environmental information during continuous adaptive navigation and target searching. At the same time, it is necessary to feed back the relative positional relationship of the target, to store and mark the target positional information. This paper uses Bayesian probability [42] to estimate the position information of the target.

The position estimation of the target for the AUV can be expressed as the following joint posterior probability density at each moment, as given in Equation (23):

(23) $$p(x_{v,k},\theta |z_{0:k},u_{0:k},x_{v,0})$$

where x represents the state of the AUV at a discrete time, θ represents the positional vector of the target, z represents the observation of the target, and u represents the control input vector.

The updates with time and measurement are given by Equations (24) and (25) respectively:

(24) $$p(x_{v,k},\theta |z_{0:k-1},u_{0:k},x_{v,0})=\int p(x_{v,k}|x_{v,k-1},u_{k})p(x_{v,k-1},\theta |z_{0:k-1},u_{0:k-1},x_{v,0})dx_{v,k-1}$$

(25) $$p(x_{v,k},\theta |z_{0:k},u_{0:k},x_{v,0})=\frac{p(z_{k}|x_{v,k},\theta )p(x_{v,k},\theta |z_{0:k-1},u_{0:k},x_{v,0})}{p(z_{k}|z_{0:k-1},u_{0:k})}$$

The information of all targets is included in the database, and the most accurate observations are saved after the comparison and fusion of the data as given in Equation (26):

(26) $$X_{\Omega }=\min\{X_{\Omega ,i,j},i=1\ldots n,j=1\ldots m\}$$

where $X_{\Omega }$ is the observational error of the target position, i is the target number, and j is the number of observations.

**Figure 4:**
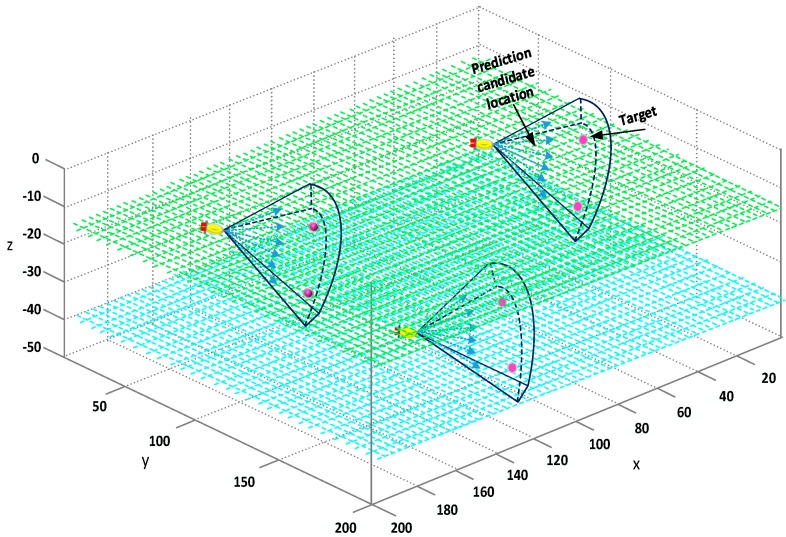
Location prediction.

AUV predicts several new candidate locations at each time step. For every candidate position, it corresponds to an independently estimated value from standard optimization function. Among all the estimated values calculated by the standard optimization function, the ${\hat{J}}_{k}$ of the optimal estimation is taken as the new position of the AUV at the next moment, as shown in Figure 4.

The choice of a good prediction position for AUV ensures that ${\hat{J}}_{k}$ is a reliable and accurate estimation of the optimization criteria function J. Using the UKF filter for the new position predicted by the AUV can reduce the impact of nonlinear interference, and ensure that the AUV can operate according to the preset trajectory. By continuously updating and correcting the probability information of observations through Bayesian estimation, the confidence interval and positioning accuracy of the target positioning can be improved.

## 5. Simulation Research

In this paper, there is a collaborative experiment for target searches in a 3D environment. There are five AUVs in the search area. Static targets and dynamic targets are randomly set. The situation of obstacles is not considered in this experiment. The state of AUV is determined by the equation of motion and the searching strategy. Assume that the AUV can detect the target through the forward-looking sonar and recognize that the target appearing in the sonar is a dynamic target or a static one. All AUVs and dynamic targets are limited to move within a given mission. The AUV records all information for observed static targets in a three-dimensional environment. The dynamic targets randomly appear, and they move according to the moving equation. When the dynamic target enters the AUV sight range, it is detected. However, according to the positioning requirements, the positioning accuracy must be ensured before it can be defined as the target within the field of view as an effective target.

In order to prove the effectiveness of the adaptive prediction algorithm in a completely unknown 3D underwater environment, this experiment was performed in MATLAB. In order to reduce the time cost of the experiment, the size of the target searching area is set to 200 × 200 × 50, and the task area is divided into five layers. Each layer is defined as a two-dimensional array matrix of [200, 200]. Five AUVs perform searching missions. Each AUV has a sonar sight of 20 m. Experiments include a static target search, a random dynamic target search, and a coordinated search following an AUV failure.

### 5.1. Static Target Search

**Figure 5:**
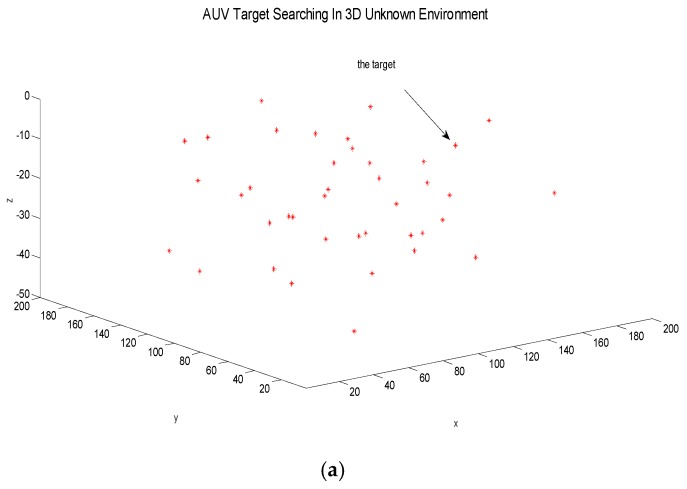

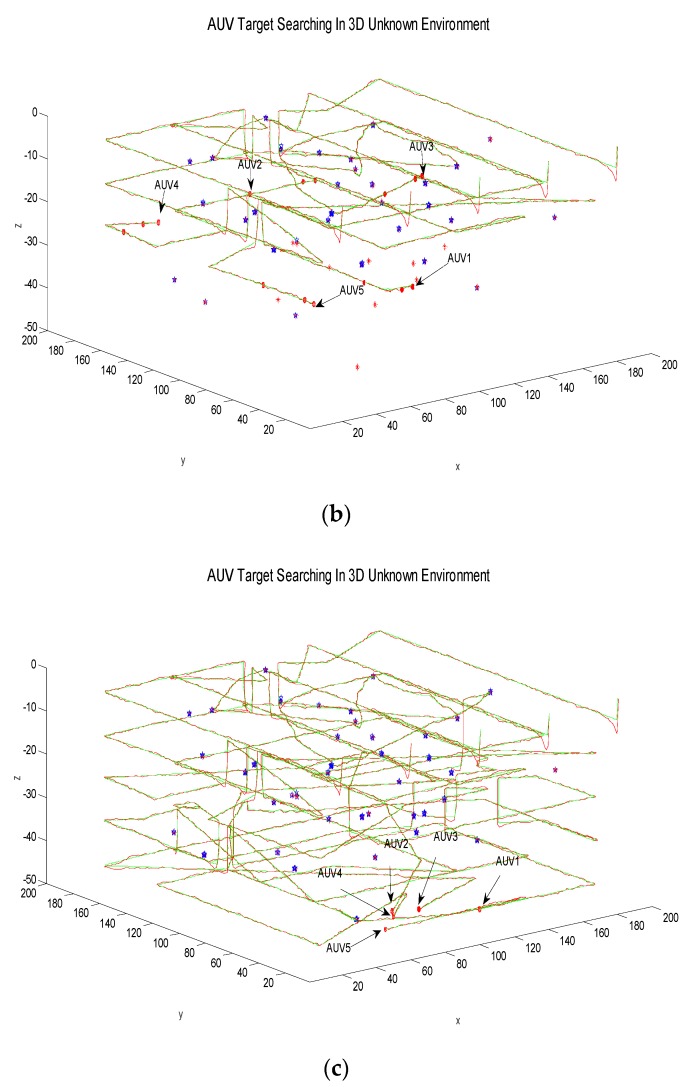
Static target search of the autonomous underwater vehicles (AUVs). (**a**) Initial state; (**b**) Search process with static targets; (**c**) The AUV’s last search results.

All targets in the three-dimensional underwater environment are assumed to be static ones, in order to verify the basic characteristics of the adaptive prediction algorithm. The initial positions of the five AUVs are (20,0,0), (60,0,0), (100,0,0), (140,0,0) and (180,0,0). The initial information for all static targets is randomly set and the location information is unknown. Static targets are randomly arranged as shown in Figure 5a.

Each AUV continuously perceives the surrounding environment through forward-looking sonar, and uses the adaptive dynamic prediction searching algorithm to generate the track for each AUV. This is because the static target and other characteristic information cannot appear in the visual field of AUV at all times; that is, the AUV may experience a no sense data situation. In view of this situation, the large-scale operation area is divided into several sub-areas. This paper combines the sub-area searching strategy with the adaptive searching algorithm. By alternately switching between the two modes of state with and without target, it is guaranteed that each AUV can independently respond to different situations in the unknown environment by using an adaptive predictive searching algorithm. The experiment counts the situation in which the target positioning accuracy is satisfied in the forward-looking sonar, and the total cost for the searching task of the five AUVs is 9995. The specific process is as follows: Figure 5b shows the process of a collaborative target search for multiple AUV. At the beginning, five AUVs are in the same layer, and then the AUVs use the forward-looking sonar to locate the target. There are some positioning errors during the process. After multiple positioning, the red target will become blue when it meets the positioning accuracy. This process indicates that the target observation position has been predicted successfully. It can be seen from Figure 5b that AUV2, and AUV3 are in the water layer 10 m underwater to perform the target search task, and that AUV1, 4, and 5 have appeared in the layer 25 m underwater to perform the task. The red line represents the actual trajectory of the AUV, and the green line represents the online designed trajectory that is based on the optimization function and UKF filtering. The reason for the above-mentioned AUV positioning errors is that GPS positioning will fail in a real underwater environment. So, if only the inertial navigation sensor is used, AUVs cannot achieve precise positioning. Due to the accumulation of positioning errors, there will be positional deviations during the final long-term navigation. Figure 5c shows the final search results for multiple AUVs. At this time, five AUVs have completed all target search tasks in the unknown environment, and so they will stop performing tasks.

**Figure 6:**
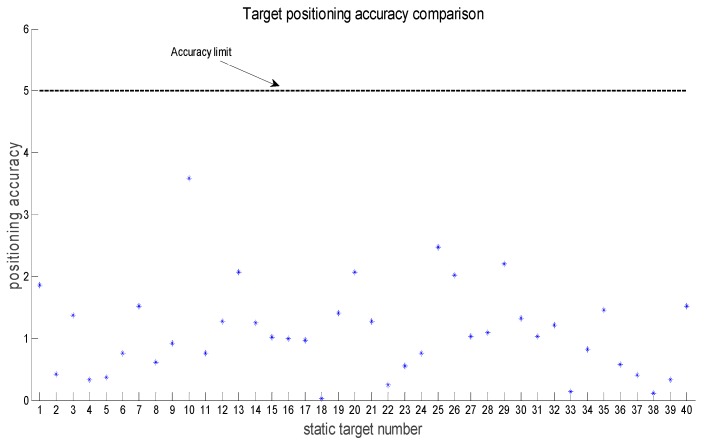
Targets positioning results.

Due to the relative distance between the AUV and the target being inversely proportional to the positioning accuracy, the positioning requirement was satisfied when the target appears in the view field of AUV and the relative distance is not higher than 5 m. Therefore, in this collaborative searching process, the positioning of each target is shown in Figure 6.

### 5.2. Random Dynamic Target

**Figure 7:**
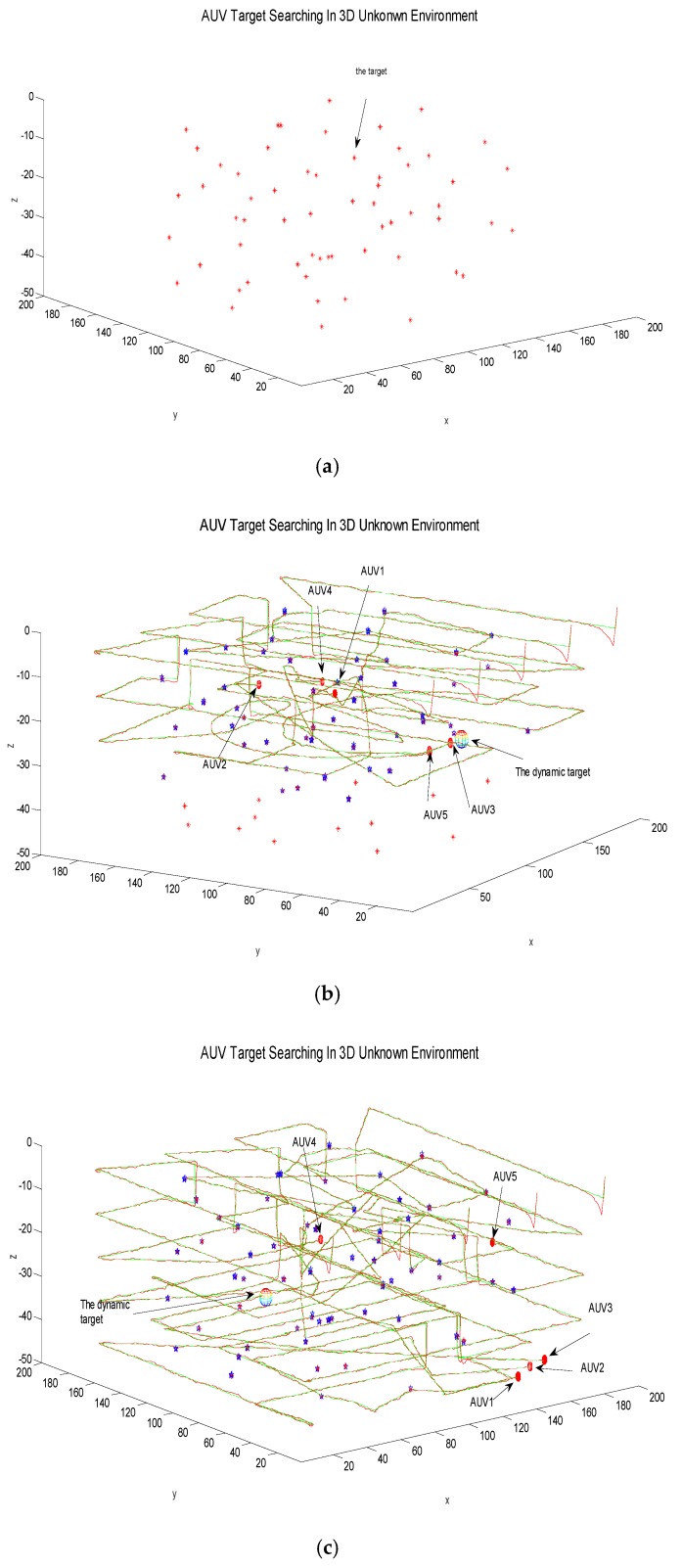
Random dynamic target search of the AUVs. (**a**) Initial state; (**b**) Search process with static targets and a random dynamic target; (**c**) AUV’s last search results.

The second simulation experiment tests the case of the existence of both static and dynamic targets, which is more complicated than the case with only static targets. In the second case, the static targets are randomly set, and the dynamic targets appear randomly in the three-dimensional environment. A regular uniform turning motion is performed at a certain depth plane. The AUV can identify dynamic targets and static targets based on the forward looking sonar, and the experimental process of target searching using adaptive searching algorithm is shown in Figure 7.

In the course of target searching for the AUV, the dynamic target appears in the third searching layer and is first discovered by AUV3. The AUV3 can notify the nearest AUV in the AUV team and round the dynamic target together, which is shown in Figure 7b and the notified unit is AUV5. At this point, the task sub-areas that AUV3 and AUV5 do not complete will be re-assigned to the other three AUVs, and the task will be continued. Therefore, this method can deal with the case of random occurrence of dynamic targets in an unknown environment.

### 5.3. Some AUVs Break Down

**Figure 8:**
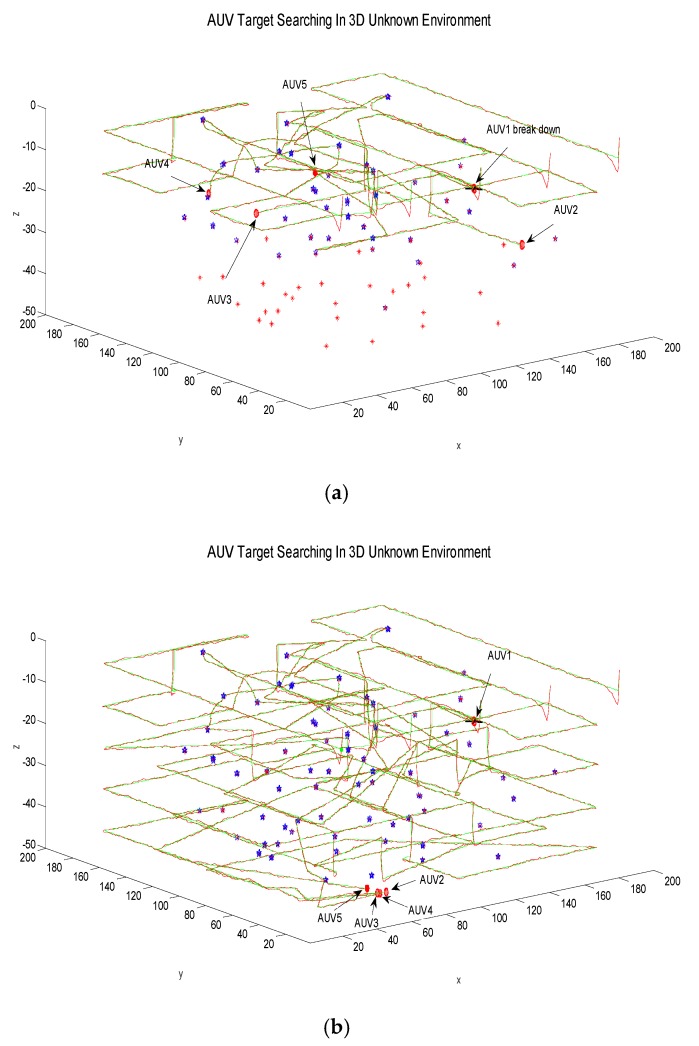
Search process when one AUV breaks down. (**a**) One AUV breaks down; (**b**) The AUV’s last search results.

When operating in an actual three-dimensional underwater environment, one or more AUVs may break down. In the third experiment, all random static targets are rearranged. Now let an AUV fail at random and stop the target searching. At the same time, the task sub-area that the failed AUV has locked in this search layer is canceled. The task assignment is re-executed by the remaining AUVs, and all target search tasks of other search layers are coordinated, which is described in Figure 8.

The Figure 8 shows that AUV1 has failed during the collaborative search of five AUVs. The task of AUV1 in other target sub-areas locked in this search layer is then cancelled. The task sub-area allocation is recalculated and assigned to the remaining AUVs. The final searching results in Figure 8b show that under certain AUV failure conditions, the adaptive predictive searching algorithm utilizes the interactive information inside multiple AUVs, adjusts the target searching strategy, and it finally completes the multi-AUV collaborative target search task efficiently.

### 5.4. Comparison of Different Algorithms

**Figure 9:**
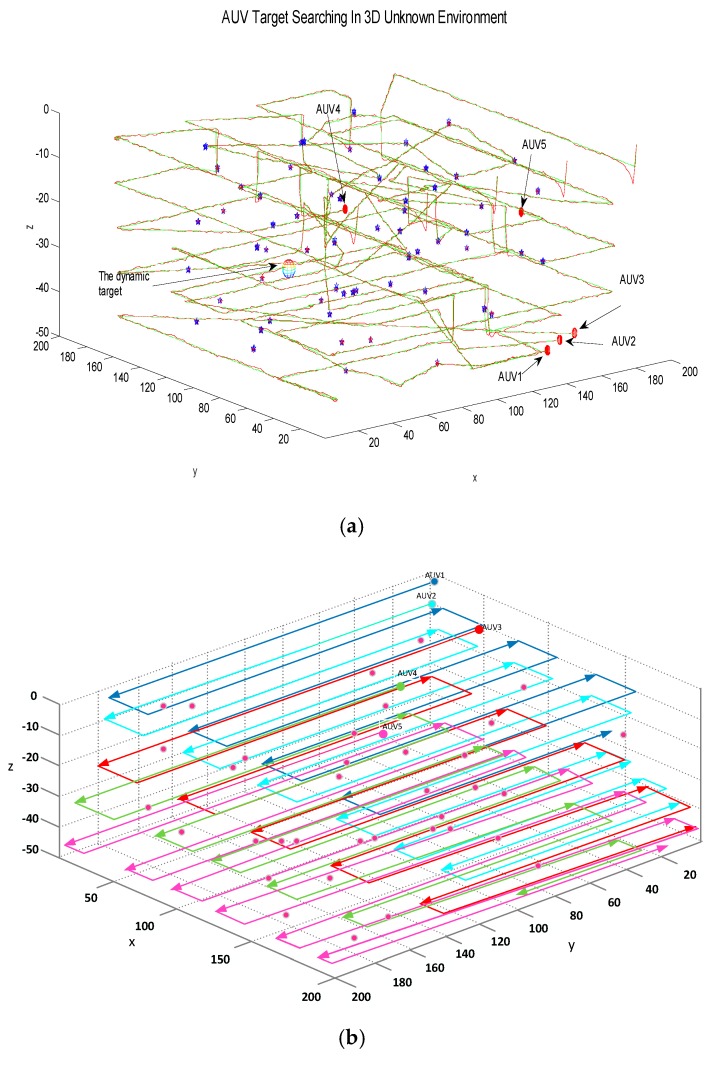

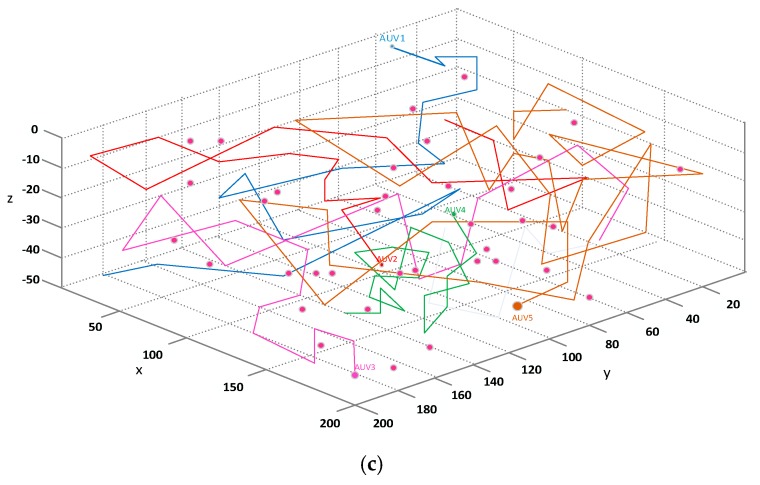
The cost of three search algorithms. (**a**) Adaptive prediction search algorithm; (**b**) Lawn-mowing algorithm; (**c**) Random algorithm.

The proposed algorithm is expected to improve the efficiency for target search in the 3D underwater environments compared with other commonly used algorithms. The random algorithm [43] randomly selects any one of the prediction points for further movement until they finally meet all targets. The lawn-mowing algorithm [25] can alternately cover the entire work area from one end to the other before detecting the target. The searching process using these three different algorithms in the same environment is depicted in Figure 9.

In this comparison simulation environment, there are only static targets of the same number, and the initial positions of all the targets are randomly assigned. Figure 9 shows the search process of three different algorithms. From the results in Figure 9, compared with the other two algorithms, the proposed algorithm can achieve the shortest search path for each AUV or multiple AUVs under the premise of hierarchical search. Most optimization algorithms will lose their meaning in the same target searching task when the AUV does not know any environmental information in advance. Also, there are other problems, such as the inability to achieve the target’s positioning requirements, large positioning errors, or missing key target information. The lawn-mowing algorithm uses a pre-set rigid search path, which cannot be adjusted based on changes in environmental information; therefore it commonly requires a longer search path than the proposed algorithm. The random algorithm may drive the AUV to repeatedly search in the same area due to the random motion of the guided AUV, thereby also increasing the length of the searching path.

In order to further compare the performance of different search algorithms, this paper investigates and compares the total generation value of the number of preset targets when using different algorithms. The end condition of the experiment is to search the same number of targets without any AUV failure. The experiment only involves static targets and there are five AUVs and 200 × 200 × 50 workspaces. The assumed targets and the initial position of the five AUVs are randomly distributed. Compared with traditional algorithms, the results of simulation show that the proposed algorithm can guide multi-AUV to complete target search task efficiently and adaptably in the unknown underwater environment. The search cost values of the target searching experiment process with three different algorithms are listed in Table 1.

**Figure 10:**
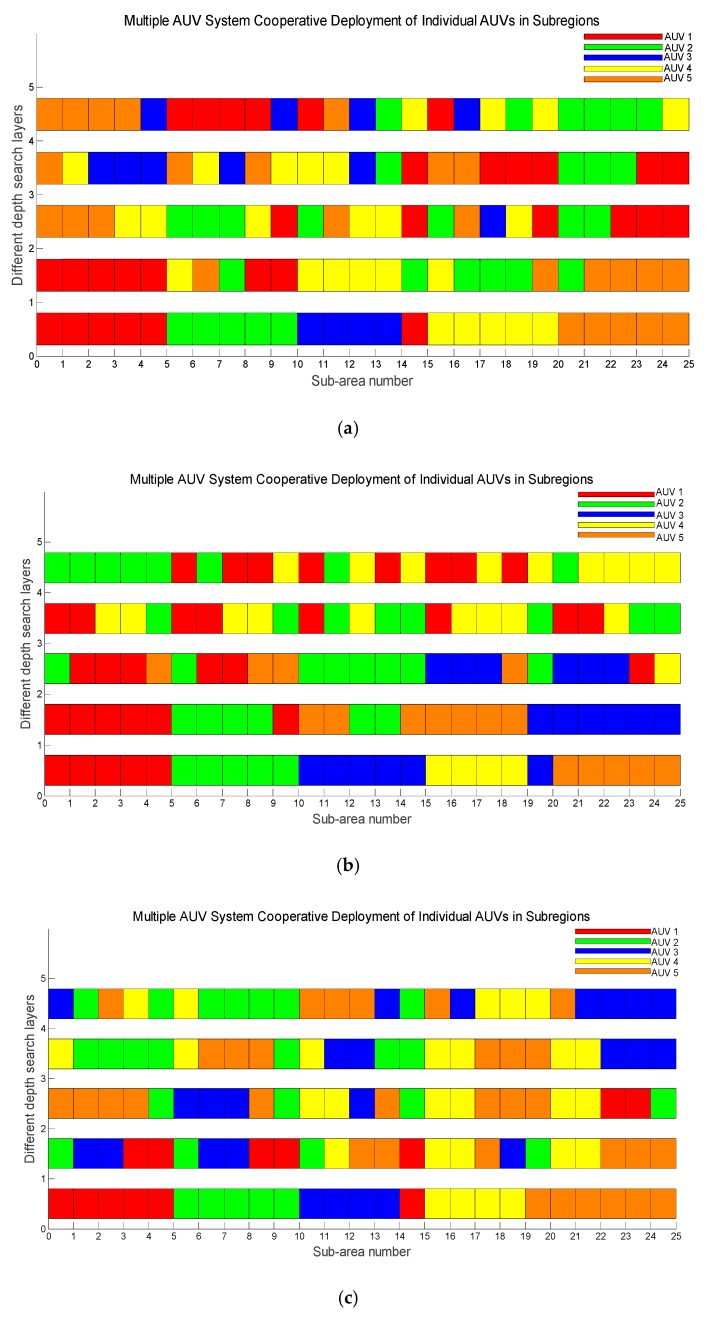
Self-adaptive allocation of individual AUVs among sub-regions in a multi-AUV system under an adaptive predictive search algorithm. (**a**) Collaborative deployment scheme under static targets. (**b**) Collaborative deployment scheme under dynamic targets. (**c**) Collaborative deployment scheme under faulty AUVs.

In Figure 10, the map of the coordinated task area of multiple AUV systems in three unknown environments is given. They are the static target search of multiple AUV systems in an unknown environment, the searching and tracking of dynamic targets, and the target searching of failed AUVs.

The specific collaborative search tasks are shown in Table 2.

Among them, the numbers in Table 2 represent the numbers of each AUV in the multiple AUV system.

From the simulation results, as shown in Table 1, Figure 10, and Table 2, the following conclusions can be made.

1. In the three strategies, the proposed algorithm exhibits the highest efficiency, and the random algorithm presents the lowest efficiency. At the end of the traversal of all sub-areas in an unknown underwater environment, the proposed algorithm only takes 15–25% of searching time compared to the average search cost of the random algorithm.
2. When the number of targets increases, the average search cost of the adaptive predictive search algorithm proposed in this paper is significantly reduced, while the average search cost of the random search algorithm increases. Therefore, it can be concluded that the algorithm proposed in this paper is more efficient in the case of an increase in the number of targets.
3. Compared with the traditional algorithm, whether it is the complexity of the search environment or the coordinated task deployment of multiple AUVs, the proposed algorithm is the most stable and the most efficient one.

## 6. Conclusions

This paper presents an adaptive prediction algorithm for cooperative target search tasks of multi-AUV. First, the individual AUV perceives its surroundings through the forward-looking sonar. Considering the influence of underwater communication distance and bandwidth limitation, all AUVs are in the communication range exchange the searching situation of the allocated sub-areas. Then, based on the established mathematical models of motion and sonar, the adaptive predictive search algorithm and task allocation strategies of sub-region segmentation are used to complete the multi-AUV collaborative target search task in the unknown underwater environment. In this case, all of the location information of targets searched by AUV is marked. The simulation results show that the proposed algorithm can enable the multi-AUV system to complete the target search task efficiently under the unknown underwater environment with all targets set randomly. Moreover, in the case of an AUV failure, it also can ensure that other AUVs cooperate to complete the remaining target search tasks. Simultaneously, the proposed algorithm can improve the efficiency of the target search in an unknown underwater environment, and it still has great potential for future development and expansion. For example, due to the influence of ocean currents, AUV will have errors in the positioning and searching of targets. It is still necessary to continue to study this issue.

## Figures and Tables

**Table 1:** Comparison of the cost of the search method.

Target Number	Adaptive Prediction Method	Lawn-Mowing Algorithm	Random Algorithm
30	8465	20,000	45,713
40	9995	20,000	58,505
50	7550	20,000	51,214

**Table 2:** Subregion allocation at each search level.

Areas	First Search Layer	Second Search Layer	Third Search Layer	Fourth Search Layer	Fifth Search Layer
Area 1	1	1	2	3	3
Area 2	1	3	3	5	3
Area 3	1	3	1	1	3
Area 4	1	3	2	1	1
Area 5	1	3	1	1	1
Area 6	2	1	4	4	2
Area 7	2	1	3	2	1
Area 8	2	1	5	5	3
Area 9	2	1	5	5	1
Area 10	2	3	2	1	5
Area 11	3	4	4	3	4
Area 12	3	4	3	4	2
Area 13	3	4	1	2	3
Area 14	3	4	1	2	1
Area 15	3	4	1	5	5
Area 16	4	5	4	3	2
Area 17	4	5	3	4	2
Area 18	4	5	3	3	2
Area 19	4	5	5	2	1
Area 20	4	5	2	1	5
Area 21	5	4	4	3	4
Area 22	5	2	4	4	4
Area 23	5	3	4	4	4
Area 24	5	5	5	2	5
Area 25	5	2	5	5	5
